# Safety and Efficacy of Apixaban vs Warfarin in Patients With Stage 4 and 5 Chronic Kidney Disease: A Systematic Review

**DOI:** 10.7759/cureus.30230

**Published:** 2022-10-12

**Authors:** Hameeda Fatima, Ijeoma Nwankwo, Mahvish Anam, Shrinkhala Maharjan, Zainab Amjad, Abdelrahman Abaza, Advait M Vasavada, Akhil Sadhu, Carla Valencia, Safeera Khan

**Affiliations:** 1 Internal Medicine, California Institute of Behavioral Neurosciences & Psychology, Fairfield, USA; 2 Internal Medicine, Dow Medical College, Dow University of Health Sciences, Dr. Ruth K.M. Pfau, Civil Hospital Karachi, Karachi, PAK; 3 Research, California Institute of Behavioral Neurosciences & Psychology, Fairfield, USA; 4 Pathology, California Institute of Behavioral Neurosciences & Psychology, Fairfield, USA; 5 Internal Medicine, Meghji Pethraj Shah Medical College, Jamnagar, IND; 6 Emergency Medicine/Plastic Surgery, All India Institute of Medical Sciences (AIIMS) New Delhi, New Delhi, IND; 7 Family Medicine, California Institute of Behavioral Neurosciences & Psychology, Fairfield, USA

**Keywords:** dialysis, stroke prevention, risk of bleeding, venous thromboembolsim, non valvular atrial fibrillation, direct oral anticoagulant therapy, chronic kidney disease (ckd), end stage renal disease (esrd), apixaban, warfarin

## Abstract

Warfarin has been an anticoagulant of choice in patients with advanced Chronic Kidney Diseases (CKD) at stages 4 and 5 for decades, but with the advent of Novel Oral Anticoagulants (NOACs), there has been a sharp rise in their prescriptions. Among all NOACS, apixaban is the least reliant on kidney function and is a very popular choice for this patient population. However, being utilized extensively, most of the landmark trials evaluating the safety and efficacy of apixaban excluded patients with Creatinine Clearance (CrCl) <25mL/min/1.73 m^2 ^or Serum Creatinine (SCr) *≥*2.5mg/dL. Its approval for advanced CKD patients came from limited pharmacokinetic data only. We conducted a systematic review comparing the safety and efficacy of apixaban to warfarin in patients with stage 4 and 5 CKD and on dialysis.

We queried major research literature databases, including MEDLINE, PubMed, PubMed Central (PMC), Cochrane Central, and ScienceDirect to find relevant articles without any time or language restrictions. After screening and quality checks, we identified 11 studies relevant to our research question, of which nine were retrospective cohort studies, one was a post-hoc analysis of a randomized controlled trial (RCT), and one was an RCT. The included studies had a total of 27,007 patients, with 4,335 patients taking apixaban and 22,672 on warfarin. The results indicate that the overall efficacy of apixaban was equivalent to warfarin for the prevention of stroke, systemic embolization, and recurrent venous thromboembolism, but apixaban showed an equivalent and, in some studies, better safety profile than warfarin concerning the occurrence of bleeding. Apixaban may hence be considered a reasonable alternative to warfarin in patients with Stage 4 or 5 CKD and receiving dialysis. In light of the reviewed articles, we conclude that apixaban has similar efficacy and somewhat superior safety profile to warfarin, with more randomized controlled trials required to add to the evidence.

## Introduction and background

Chronic Kidney Disease (CKD) is described as having kidney damage or an estimated Glomerular Filtration Rate (eGFR) less than 60 ml/min/1.73 m^2^, persisting for three months or more, irrespective of the cause [1].The five stages of CKD as classified according to Glomerular Filtration Rate (GFR) are Stage 1: GFR (>90 mL/min/1.73m^2^), Stage 2: GFR (60-89 mL/min/1.73 m^2^), Stage 3a: GFR (45-59 mL/min/1.73 m^2^), Stage 3b: (30-44 mL/min/1.73 m^2^), Stage 4: GFR (15-29 mL/min/1.73 m^2^) and Stage 5: (GFR < 15 mL/min/1.73 m^2^ or dialysis) [2]. Advanced Chronic Kidney Disease (ACKD) includes stages 4 and 5 of the CKD classification. Overall, CKD affects about 13.4% of the global population owing to almost 843.6 million individuals, with 0.4% having stage 4 and 5 and 0.1% having stage 5 CKD, respectively [3].

Compared to the general population, advanced CKD patients are at an increased risk of Atrial Fibrillation (AF) and Venous Thromboembolism (VTE), warranting short and long-term anticoagulation treatment [4-6]. Advanced CKD patients are also at an increased risk of bleeding, making it difficult to choose an optimal anticoagulant agent for this population [5]. Historically, warfarin has been the cornerstone of therapy for patients requiring anticoagulation with stage 4 and 5 CKD [7]. But due to frequent monitoring to maintain the therapeutic range of the International Normalization Ratio (INR), i.e., 2-3 and multiple foods and drug interactions, the development of alternate and better oral anticoagulation agents with less frequent monitoring was vital [8-10].

Novel Oral Anticoagulants (NOACs) were introduced into the market in October 2010. Given as fixed doses without routine coagulation monitoring, these agents are at least as effective and more convenient to administer than traditional anticoagulants [11]. Owing to their ease of administration and monitoring, NOACs gained significant popularity quickly. As a result, the number of prescriptions for NOACs increased dramatically [12]. Among NOACS, apixaban, a direct Factor Xa inhibitor, was approved by the United States (US) Food and Drug Administration (FDA) in 2012 for use in patients with non-valvular atrial fibrillation and in 2014 to treat Deep Venous Thrombosis (DVT) and Pulmonary Embolism (PE) [13].

Several landmark trials were undertaken to study the safety and efficacy of apixaban, including ARISTOTLE (Apixaban for Reduction in Stroke and Other Thromboembolic Events in Atrial Fibrillation) and AMPLIFY (Apixaban for the Initial Management of PE and DVT as First-Line Therapy) comparing apixaban with warfarin in AF and acute VTE, but their major limitation was that they excluded CKD patients with Creatinine Clearance (CrCl <25mL/min/1.73 m^2^ or Serum Creatinine (SCr) 2.5mg/dL [14,15]. Still, in 2014, apixaban was approved by the US FDA for End Stage Renal Disease (ESRD) patients with AF based on limited pharmacokinetic data only [16].

Among all NOACS, apixaban is the least reliant on kidney function for clearance, as only 25% of the drug is eliminated by the kidneys. It is also minimally affected by dialysis in that a four-hour dialysis session (Opti flux F180NR dialyzer, dialysate flow rate of 500 mL/min, blood flow rate 350 to 500 mL/min) will only remove 6.7% of the drug [17]. Despite the paucity of clinical data, apixaban is a popular drug choice in stage 4 or 5 CKD and patients on dialysis, such that it accounted for ≈25% of new anticoagulation prescriptions for patients with ESRD in 2015 [18].

The aim of conducting this systematic review is to sum up the available good-quality evidence regarding the safe and effective use of apixaban compared to warfarin in patients with stage 4 and 5 CKD and on dialysis.

## Review

Methodology

This systemic review is reported in concordance with the Preferred Reporting Items for Systematic Reviews and Meta-Analyses (PRISMA) guidelines [19]. Data were included from previously published studies only; hence ethical approval was deemed unnecessary.

Search Strategy

An electronic search of MEDLINE, PubMed, PubMed Central (PMC), ScienceDirect, and Cochrane Central was conducted from their inception to 28 May 2022, without time or language restrictions to find the maximum quantity of articles related to the topic.

The final search strategy for PubMed, PMC, and MEDLINE is as follows: (warfarin OR coumadin OR jantoven OR vitamin K antagonist) AND (apixaban OR NOACS OR Eliquis OR factor XA inhibitor OR Direct oral anticoagulants) AND (CKD OR chronic kidney disease OR renal failure OR renal dysfunction OR dialysis). The keywords used for search in Science Direct and Cochrane Central included "Apixaban", “NOACs” “Eliquis” "Warfarin", "chronic kidney disease," "End Stage Renal Disease”, "Atrial Fibrillation", "Venous Thromboembolism” and “Stroke”.

Inclusion and Exclusion Criteria

We used the Population, Intervention, Comparison, Outcomes (PICO) model to select the studies for eligibility. The population of interest is patients with stage 4 (GFR 15-29 mL/min/1.73 m^2^) or end-stage chronic kidney disease (GFR < 15 mL/min/1.73 m^2^) and patients receiving dialysis treatments. All Randomized Controlled Trials (RCTs) and observational studies comparing apixaban vs. warfarin for anticoagulation in CKD patients were included. Patients having GFR >30mL/min/1.73 m^2^, studies comparing multiple NOACs additively to warfarin, studies not including warfarin as the control group, animal studies, case reports, editorials, expert opinions, and unpublished studies were excluded. Any duplicate studies from the same database having the same follow-up length as well as studies that did not meet the desired quality according to the quality assessment tools were also excluded.

Data Extraction and Analysis

The data from the selected studies were extracted independently by two authors and verified by a third author. A fourth author was then consulted to resolve any disparities with consensus.

Critical Appraisal of Studies

The observational cohort studies and RCTs were assessed using the New Castle Ottawa Scale and Cochrane Risk of Bias (R.OB.2) Tool, respectively [20,21]. The summary of risk assessment for selected studies is shown (Tables 1, 2) below.

**Table 1:** Cochrane Risk of Bias (R.OB.2) Tool For Randomized Controlled Trials RENAL A-F, RENal hemodialysis patients ALlocated apixaban versus warfarin in Atrial Fibrillation.

Study Name and Year	Risk of bias arising from the randomization process	Risk of bias due to deviations from the intended interventions	missing outcome data	Risk of bias in measurement of the outcome	selection of the reported result	overall bias
Stanifer et al. (2020) [22]	low risk	low risk	low risk	low risk	low risk	Low
RENAL- AF trial. (2019) [23]	low risk	some concerns	low risk	low risk	some concerns	some concerns

**Table 2:** New Castle Ottawa Scale for Observational Cohort Studies

Study name and year	Representativeness of the exposed cohort	Selection of the non-exposed cohort	Ascertainment of exposure	Demonstration that outcome of interest was not present at the start of study	Comparability of cohorts on the basis of the design or analysis	Assess the outcome	Was follow-up long enough for outcomes to occur	Adequacy of follow-up of cohorts	Total	Quality
Stanton et al. (2017) [24]	*	*	*	*	**	*	*	*	9/9	High
Hanni et al. (2020) [25]	*	*	*	*	*	*	*	*	8/9	High
Schafer et al. (2018) [26]	*	*	*	*		*	*	*	7/9	Medium
Siontis et al. (2018) [18]		*	*		*	*	*	*	6/9	Medium
Herndon et al.(2019) [27]	*	*	*	*		*	*	*	7/9	Medium
Noseworthy et al. (2019) [28]	*	*	*	*	*	*	*	*	8/9	High
Reed et al. (2017) [29]	*	*	*	*		*	*	*	7/9	Medium
Sarratt et al.(2017) [30]	*	*	*	*		*	*	*	7/9	Medium
Heleniak et al.(2020) [31]	*	*	*	*	*	*	*	*	8/9	High

Results

**Figure 1:**
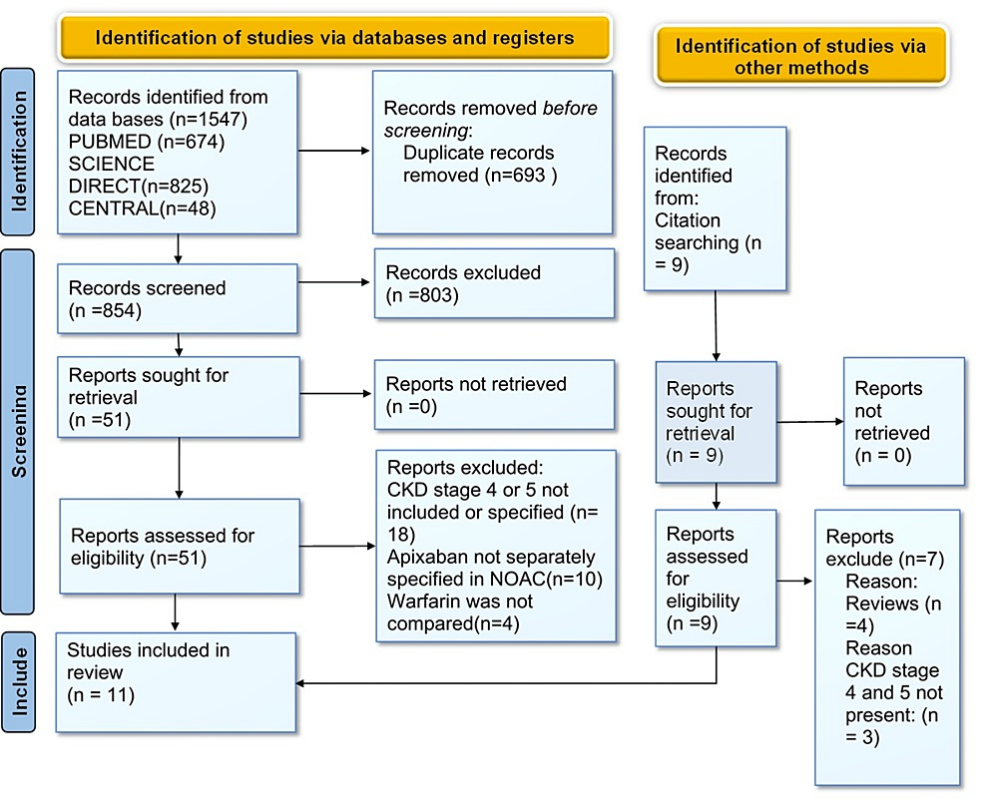
Preferred Reporting Items for Systematic Reviews and Meta-Analyses (PRISMA) flow diagram CKD, Chronic Kidney Disease; NOAC, Novel Oral Anticoagulant.

We identified a total of 1547 studies from the initial search of PUBMED, MEDLINE, ScienceDirect, and Cochrane Central databases. Identified studies were transferred to EndNote Reference Library (Version x7.5; Clarivate Analytics, Philadelphia, Pennsylvania) software, where duplicates were searched for, and 693 duplicates were removed. Eight hundred fifty-four studies were screened from titles and abstracts by two independent authors, and 803 records were excluded due to the irrelevance of the topics. Finally, 51 studies were retrieved, and their full texts and bibliographies were assessed. Nine more studies were identified from the bibliographies. All these studies were checked for eligibility and detailed inclusion and exclusion criteria. Twenty studies were further removed as they analyzed NOACs as a group, and apixaban was not separately compared; four studies failed to compare apixaban to warfarin, four studies fetched through bibliographies were reviewed, and 21 studies did not mention CKD stages 4 or 5. Finally, 11 studies were selected for the final review. The detailed PRISMA flow diagram of the included studies is shown (Figure 1) below [20].

Among the included studies, nine studies were retrospective cohorts, one was a post hoc analysis of RCT, and one was RCT. The studies totaled 27,007 patients, with 4,335 patients taking apixaban and 22,672 on warfarin. The most common indication for anticoagulation was stroke prevention for atrial fibrillation and DVT prophylaxis. The following outcomes were assessed from the selected articles involving the occurrence of Major Bleeding (MB), Clinically Relevant Non-Major Bleeding (CRNMB), minor bleeding, composite of all bleeding, stroke and systemic embolization, recurrent venous thromboembolism, and mortality. The definitions of these outcomes were used as defined in their respective studies. The general characteristics of included studies, including year and type of publication, sample size, CKD stages included, mean ages of the population, and an indication of anticoagulation, are represented (Table 3) below. 

**Table 3:** General Characteristics of Included Studies API, Apixaban; WAR, Warfarin; CKD, Chronic Kidney Disease; ESRD, End Stage Renal Disease; CrCl, Creatinine Clearance; GFR, Glomerular Filtration Rate; eGFR, Estimated Glomerular Filtration Rate; SCr, Serum Creatinine Clearance; ESRD, End Stage Renal Disease; VTE, Venous Thromboembolism; NVAF, Non-Valvular Atrial Fibrillation; AF, Atrial Fibrillation; RENAL A-F, RENal hemodialysis patients ALlocated apixaban versus warfarin in Atrial Fibrillation; ARISTOTLE Trial, Apixaban for Reduction in Stroke and Other Thromboembolic Events in Atrial Fibrillation Trial; NA, Not Available.

Study	Publication Year	Study Design	Sample Size(n)	API vs WAR	Renal Function	Mean Age (Years)	Anticoagulation Indication
Stanton et al. [24]	2017	retrospective cohort	146	73 vs 73	CrCl<25ml/min or SCr>2.5mg/dl or on dialysis	79	NVAF 73% VTE 26%
Hanni et al. [25]	2020	retrospective cohort	861	128 vs 733	CrCl<25ml/min	71	AF 41% VTE 44%
Schafer et al. [26]	2018	retrospective cohort	604	302 vs 302	GFR<29mL/min/1.73m2	72	NVAF 81%
Siontis et al. [18]	2018	retrospective cohort	9404	2351 vs 7053	ESRD on Dialysis	68	NVAF 100%
Herndon et al. [27]	2019	retrospective cohort	111	54 vs 57	eGFR<29mL/min/1.73m2 or SCr>2.5 or on dialysis	72	NVAF 69% VTE 14%
Noseworthy et al. [28]	2017	cohort study	49953	1034 vs 13987	CKD stages 4 and 5	NA	NVAF 100%
Reed et al. [29]	2018	retrospective cohort	124	74 vs 50	ESRD on Dialysis	61	NVAF 47% VTE 53%
Sarratt et al. [30]	2017	retrospective cohort	160	40 vs 120	eGFR<15ml/min undergoing chronic hemodialysis	68.7	VTE 100%
Heleniak Z et al. [31]	2020	retrospective cohort	153	61 vs 92	CKD stage 4 eGFR 15-29ml/min/1.73m2	69.5	NVAF 100%
Stanifer JW et al. [22]	2020	Post-hoc analysis of ARISTOTLE	269	136 vs 133	CrCl 25-30mL/min	81	NVAF 100%
RENAL-AF( Trial [23]	2019	Randomized Controlled Trial	154	82 vs 72	ESRD on Dialysis	69.5	NVAF 100%

Discussion

To compare the safety and efficacy of apixaban with warfarin in advanced CKD, we evaluated the prevention of stroke, recurrent VTE, and reduction in mortality as efficacy outcomes and occurrence of major, non-major but clinically significant, and minor bleeding as safety outcomes. The detailed summary and outcome variables of included studies are highlighted (Table 4) below.

**Table 4:** An Outline Summary Of Included Studies with Outcomes Of Interests and Results API, Apixaban; WAR, Warfarin; CI, Confidence Interval; HR, Hazards Ratio; GI, Gastrointestinal; IC, Intra Cranial; Diff, Difference; IS, Ischaemic Stroke; MB, Major Bleeding; CRNMB, Clinically Relevant Non-Major Bleeding; SE, Systemic Embolization; TE, Thrombo Embolism; PE, Pulmonary Embolism; DVT, Deep Venous Thrombosis; ESRD, End Stage Renal Disease; AF, Atrial Fibrillation; NVAF, Non-Valvular Atrial Fibrillation; NOAC, Novel Oral Anticoagulant; VTE, Venous Thrombo embolism; International Society on Thrombosis and Haemostasis (ISTH) criteria for major bleeding: “clinically overt bleeding accompanied by at least one of the following Fatal bleeding, and/or Bleeding in a critical area or organ, such as intracranial, intraspinal, intraocular, retroperitoneal, intra-articular or pericardial, or intramuscular with compartment syndrome, and/or Bleeding causing a fall in hemoglobin level of 20 g L^−1^ (1.24 mmol L^−1^) or more, or leading to transfusion of two or more units of whole blood or red cells”

Study		Primary Outcome of Interest and Definition used		Secondary outcomes and Definitions used		Results		Conclusion	
Stanton et al. [24]		MB (Modified ISTH)		Composite of bleeding (MB, CRNMB, minor bleeding), CRNMB (medical/surgical treatment, change in anti-thrombotic therapy), Minor Bleeding (non-major and non-CRNMB), IS (documentation in medical records on readmission and hospitalization) in NVAF patients, recurrent VTE in PE or DVT patients.		API group had less MB vs. WAR group although differences not statistically significant, no statistically significant differences in all secondary outcomes		API seems to be a reasonable alternative to WAR in severe renal impairment	
Hanni et al. [25]		Time to first bleeding or thromboembolic event (Thrombolysis in Myocardial Infarction Bleeding Criteria)		Time to 1st bleeding event, time to first thrombosis event, proportion of patients experiencing bleeding or thrombosis, severity and source of bleeding and thrombosis		Time to 1^st^ bleeding or thrombosis event was significantly diff between API and WAR groups. After controlling confounders, risk of thrombotic and bleeding events was lower in the apixaban group (hazard ratio, 0.47; 95%CI, 0.25-0.92). No statistical diff between time to, or rate of thrombosis, time to, or rate of and severity of bleeding.		API may serve as a reasonable alternative to warfarin in patients with severe renal dysfunction	
Schafer et al. [26]		Rate of MB at three months of enrolment (ISTH)		MB rates at 6 & 12 months; rates of IS (focal neurological deficit, from non-traumatic cause confirmation by chart review and ICD 9 and 10 codes) and recurrent thromboembolism(fatal or non-fatal PE, or DVT with confirmation by chart review and ICD 9 and 10 codes) at 3, 6, and 12 months		Percentage of API and WAR patients with a major bleed at 0 to 3, 3 to 6, and 6 to 12 months were 8.3% versus 9.9% (P=0.48), 1.4% versus 4% (P=0.07), and 1.5% versus 8.4% (P<0.001), respectively. There was no diff in rates of ischemic stroke or recurrent VTE at any time period.		API users had similar bleeding rates at three months compared to WAR users. However, those who continued therapy had higher MB rates with WAR between 6 and 12 months	
Siontis et al. [18]		Diff b/w groups in survival free of stroke or SE, MB; GI or IC bleeding; and death. (Bleeding considered major if associated with a critical site code (such as IC), need for blood product transfusion based on procedure code during the same admission, or death)		NA		No diff in risks of stroke/SE between API and WAR (HR, 0.88; P=0.29); API was associated with a significantly lower risk of MB (HR,0.72; P<0.001). There were significantly lower risks of stroke/systemic embolism and death with standard-dose apixaban (5 mg twice daily) compared to either reduced dose apixaban (2.5 mg twice daily; or warfarin.		Use of API may be associated with a lower risk of MB than warfarin, and a standard 5 mg twice daily dose of API was also associated with reductions in thromboembolic and mortality risks.	
Herndon et al. [27]	MB (modified ISTH)		CRNMB(clinically overt Bleeding not satisfying criteria for MB or bleeding that led to hospital admission, physician-guided medical/ surgical treatment or change in antithrombotic therapy), minor bleeding(clinically overt bleeding not meeting criteria for MB or CRNMB), composite of all bleeding and incidence of VTE or stroke.		No statistically significant diff found between API vs WAR for rates of MB and CRNMB. Statistically significant diff in minor and composite bleeding events, with more bleeding in the warfarin group. (6% vs26%, P = 0.004). composite bleeding, (20% vs 46%, P = 0.004)		it is reasonable to consider apixaban, when dosed correctly, as an alternative to warfarin.		
Noseworthy et al. [28]	Stroke and MB		NA		MB in Stage 4-5 CKD not on dialysis HR 0.43, On dialysis HR 0.29, Stroke/SE Stage 4-5 CKD not on Dialysis (HR 0.39), Stroke/SE on Dialysis (0/125). NOAC use was associated with a reduced risk of stroke without increased bleeding risk. In patients not on dialysis, NOACs were associated with similar risks of stroke, and API was associated with a lower risk of bleeding than WAR		API may be a reasonable alternative to WAR in dialysis patients		
Reed et al. [29].	Overall bleeding event rate (defined as experiencing at least one bleeding event after at least two doses of starting apixaban and at least one bleeding event after 5 days of starting warfarin)		MB (ISTH) events, CRNMB (bleeding not satisfying MB criteria or any bleeding leading to hospital admission, physician guided Medical/surgical treatment, or change in antithrombotic therapy), minor bleeding (all other bleeding), recurrent VTE in patients treated for DVT or PE, and IS (new neurologic deficit and imaging Confirmation) in NVAF patients.		API group had significantly fewer overall bleeding events than the warfarin group (18.9% vs. 42.0%; P = .01). MB events less frequent in API vs. warfarin group (5.4% vs. 22.0%; P = .01). No recurrent ischemic strokes in either group. A lower, non-significant, incidence of recurrent VTE found in API group vs WAR group (4.4% vs 28.6%; P = .99)		Compared to warfarin, apixaban is a safe and effective alternative in patients with ESRD maintained on dialysis, with apixaban patients experiencing fewer bleeding events than warfarin.		
Sarratt et al. [30].	MB (ISTH)		CRNMB (bleeding causing hemodynamic compromise, hospitalization, unexpected hematoma or excessive wound hematoma, epistaxis, hemoptysis, haematuria, gingival, GI, or rectal bleeding, and any other bleeding leading to intervention) and minor bleeding.		7 MB events in WAR group vs zero in API group (P = 0.34). Similar rates of CRNMB events (12.5% vs 5.8%, P = 0.17) and minor bleeding (2.5% vs 2.5%, P = 0.74) events in patients receiving apixaban and warfarin		No statistically significant diff in bleeding rates in patients receiving API vs. WAR. Apixaban may be a cautious consideration in hemodialysis patients until further evidence.		
Heleniak et al. [31]	IS or Transient Ischemic Attack (TIA) occurrence		MB (ISTH), CRNMB (hemorrhage not fulfilling MB criteria, required medical intervention, led to hospitalization, or prompted face-to-face evaluation)		No statistically significant diff observed between API and WAR in terms of MB, CRNMB, TE, and mortality.		Apixaban and rivaroxaban at reduced doses display similar effectiveness and safety compared to warfarin, but they are more convenient.		
Stanifer et al. [22]	Time to first MB(ISTH), ischemic or hemorrhagic stroke, or SE		Composite of MB or CRNMB (ISTH), death from any cause, cardiovascular death, and myocardial infarction.		API caused less MB (HR, 0.34 [95% CI, 0.14–0.80]) and major or CRNMB (HR, 0.35 [95% CI, 0.17–0.72]) vs WAR. No statistically significant diff found between HRs for stroke or SE.		Among patients with AF and CrCl 25 to 30 mL/min, API caused less bleeding than WAR.		
Renal AF 2019 US trial. [23]	MB and CRNMB (ISTH)		Stroke or SE		API VS WAR MB:8.5% vs. 9.7%, CRNMB:31.5% vs. 25.5% (p > 0.05) Stroke:2.4% vs. 2.8% Mortality:25.6% vs 18.1% (HR:1.47 95%CI: 0.74 to 2.93)		API 5 mg BID results in similar rates of bleeding and strokes as warfarin among patients with ESRD on hemodialysis		

Risk of Bleeding

None of the studies in this systematic review reported inferior safety of apixaban as compared to warfarin. Apixaban was either similar to or superior to warfarin in the context of causing a bleeding event. Stanton et al. found no statistically significant differences between apixaban and warfarin with regard to bleeding, including major bleeding (9.6% vs. 17.8%, p=0.149), clinically relevant nonmajor bleeding (11% vs. 8.2%), minor bleeding (1.4% vs. 2.7%), and a composite of all bleeding (21.9% vs. 27.4%, p=0.442), although they reported lower rates of bleeding in apixaban arm with statistically insignificant differences [24]. In the Herndon et al. study of 111 veterans with advanced CKD, no statistically significant differences were found between apixaban and warfarin groups in terms of major bleeding (7% vs. 14%, p=0.362) and clinically relevant nonmajor bleeding (5% vs. 7%, p = 0.712) [27].

In another study on 160 patients receiving chronic hemodialysis, Sarratt et al. noted similar findings with no difference in apixaban vs warfarin with regards to rates of major bleeding (0% vs 5.8, p=0.338), clinically relevant non-major bleeding (12.5% vs 5.8%, p=0.166), and minor bleeding (2.5% vs 2.5%) [30]. They reported a higher percentage (12.5% vs 5.8%) of clinically relevant non-major bleeding in apixaban users, although the difference was insignificant. Noseworthy et al. also stated that among dialysis patients, there were no significant differences between apixaban and warfarin in the risks of bleeding Hazard Ratio (HR) (0.29) [28].

Heleniak et al. conducted a study on 182 patients with stage 4 CKD, with 92 patients receiving warfarin and 90 NOAC (apixaban 61, rivaroxaban 29) [31]. Within a mean follow-up of 26 months, there were no statistically significant differences between the occurrence of major bleedings or CRNMB in NOAC vs. warfarin groups (15.56% vs. 14.13%; p=0.79). They concluded that in real-life patients when warfarin therapy is closely monitored and optimized, the risk of bleeding did not differ from that among apixaban or rivaroxaban users.

The Trial to Evaluate Anticoagulation Therapy in Haemodialysis Patients with Atrial Fibrillation (RENAL-AF) showed that in apixaban vs. warfarin groups, the percentage of clinically relevant nonmajor bleeding was 31.5% vs. 25.5% (p>0.05), major bleeding was 8.5% vs. 9.7%, intracranial bleeding was 1.2% vs. 1.4% and gastrointestinal bleeding was 2.4 vs. 8.3% [23]. They concluded that apixaban 5 mg twice daily resulted in similar bleeding rates as warfarin among patients with ESRD on hemodialysis.

In our review, the studies also reported superior safety profiles for apixaban vs. warfarin in terms of risk of bleeding. Noseworthy et al. noted that apixaban was associated with a lower risk of bleeding than warfarin in patients with advanced kidney disease not receiving dialysis, HR 0.43 (0.27, 0.68) [28]. Herndon et al. study involving advanced CKD veteran patients also found a statistically significant difference between apixaban vs. warfarin when comparing minor bleeding (6% vs. 26%, p=0.004) and composite bleeding (20% vs. 46%, p=0.004) events, with less bleeding in the apixaban group [27].

Schafer et al. compared bleeding events between apixaban and warfarin in a matching cohort of 604 patients with advanced CKD according to the time of occurrence of outcomes [26]. They assessed bleeding outcomes at three, six, and 12 months respectively, and found that during the first six months, both groups had similar bleeding rates, but apixaban showed significantly lower bleed rates compared to warfarin Odds Ratio (OR, 0.16) when observed for up to 12 months. Siontis et al. studied 25,523 Medicare beneficiaries with ESRD undergoing dialysis. In the matched cohorts, they found that apixaban was associated with a significantly lower risk of major bleeding when compared to warfarin (HR, 0.72; 95% CI, 0.59-0.87; p<0.001) [18].

Stanifer et al. compared apixaban’s safety and efficacy in 269 patients with atrial fibrillation and advanced chronic kidney disease with CrCl 25 to 30 mL/min enrolled in the ARISTOTLE trial. They found that apixaban resulted in a lower relative risk of major bleeding (3.78 vs 11.94 events per 100 patient-years; HR, 0.34 {95% CI, 0.14-0.80}) when compared with warfarin [22]. It also resulted in lower risk rates of major or CRNM bleeding (5.43 versus 16.75 events per 100 patient-years; HR=0.35 {95% CI, 0.17-0.72}) when compared with warfarin. Consistent with findings from the overall ARISTOTLE trial, in the subgroup of patients with atrial fibrillation and CrCl 25 to 30 mL/min, apixaban resulted in less bleeding than warfarin, including lower rates of major bleeding.

Hanni et al. found a significant difference in time to the first bleeding or thrombotic event between the apixaban and warfarin groups [25]. When atrial fibrillation and coronary artery bypass grafting were controlled as confounders, the risk of thrombotic and bleeding events was less in the apixaban group (HR=0.47; 95% CI, 0.25-0.92). There was no statistical difference between groups in time to bleeding (46 days vs. 54 days, p=0.886), the severity of bleeding, and the rate of bleeding (5.5% vs. 10.9%, p =0.06). Overall, a decrease in composite risk of bleeding or thrombosis was observed in patients receiving apixaban vs. warfarin.

Risk of Stroke/Systemic Embolization, Recurrent VTE, and Mortality

Taken together with all the studies, the efficacy of apixaban in preventing stroke/systemic embolization (SE), recurrent VTE, and mortality was similar to warfarin. The study of Stanton et al. found no significant difference in relation to stroke prevention in non-valvular atrial fibrillation (NVAF), as the percentage of stroke was similar in apixaban vs. warfarin groups, 7.5% vs. 7.5%, and none of the patients in their study experienced recurrent VTE in both groups 0%vs 0% [24]. Herndon et al. also did not report any differences between apixaban and warfarin groups in terms of stroke or VTE rates in advanced CKD patients [27]. In the study of Stanifer et al., the comparisons between apixaban versus warfarin for stroke or systemic embolism yielded no differences (HR=0.55 {95% CI, 0.2-1.5}) [22].

Noseworthy et al. also documented similar findings in a seven-year United States administrative database study, with no significant differences between rates of stroke and systemic embolization between apixaban and warfarin groups in patients not receiving dialysis, HR=0.39 (0.12, 1.22) [28]. In patients receiving dialysis, no stroke or SE events were noted in the apixaban group when compared to warfarin.

In the study of Schafer et al., ischemic stroke and recurrent VTE outcomes were assessed at three, six, and 12 months respectively [26]. During the zero to three, three to six, and six to 12 months follow-up periods, the stroke rates were similar, with p values of 1 in each comparison. The rates of thromboembolism also did not differ between the two groups in all the defined periods, with p values of 1, 0.5, and 0.5 for zero to three, three to six, and six to 12 months, respectively. Reed et al. reported that the patients taking apixaban had a lower but non-significant VTE recurrence than the warfarin patients (4.4% vs. 28.6%, p=.99) [29]. In their study, no patients in either the apixaban group or the warfarin group suffered from an ischemic stroke.

Heleniak et al. conducted a study on 182 patients with stage 4 CKD and atrial fibrillation, with 92 patients receiving warfarin and 90 NOAC [31]. Among the NOAC group, 61 were taking apixaban and 29 rivaroxaban. Within a mean follow-up of 26 months, arterial thrombo-embolism occurred in 12.2% of subjects on NOAC and 7.61% on warfarin, differences being statistically insignificant with a p-value of 0.30.

In the Siontis et al. study of Medicare beneficiaries with ESRD on dialysis, the matched cohorts displayed no difference in the risks of stroke/systemic embolism between apixaban and warfarin (HR=0.88; 95% CI, 0.69-1.12; p=0.29) [18]. A sensitivity analysis compared standard dose apixaban (5 mg twice daily) with reduced dose apixaban (2.5 mg twice daily) and warfarin. It was found that the standard-dose apixaban resulted in significantly lower risks of stroke/systemic embolism and death when compared with reduced dose apixaban (HR=0.61; p=0.04 for stroke/systemic embolization; HR=0.64; p=0.01 for death) and warfarin (HR=0.64; p=0.04 for stroke/systemic embolization; HR=0.63; p=0.003 for death) [14]. They concluded that a standard 5 mg dose of apixaban was not only associated with reduced thromboembolic events but also a reduction in mortality compared to warfarin.

Hanni et al. found no statistical difference between apixaban and warfarin groups for time to thrombosis (83 days vs. 54 days, p=.648), rate of thrombosis (5.5% vs. 10.3%, p=.08), and the severity of thrombotic mortality also revealed no significant difference between warfarin and apixaban groups (14.9% vs. 11.8% p=.72) [25]. The time to the first event, either bleeding or thrombosis, was significantly different between the apixaban and warfarin groups. When atrial fibrillation and coronary artery bypass grafting were controlled as confounders, the apixaban group had a lower risk of thrombotic and bleeding events when compared to warfarin(HR= 0.47; 95% confidence interval, 0.25-0.92).

In the Renal AF trial of 154 ESRD patients on dialysis, the percentages of stroke/systemic embolization were 2.4% vs. 2.8%, and cardiovascular death was 11% vs. 5.6% of the patients [23]. There was no statistically significant difference between overall mortality in apixaban vs. warfarin groups (25.6 %vs. 18.1%, HR=1.47; 95%CI 0.74- 2.93). In conclusion, this trial indicated that apixaban 5 mg twice daily resulted in similar rates of strokes as warfarin among patients with ESRD on hemodialysis.

Limitations

There are certain limitations to this study. Overall, the number of patients receiving apixaban was much less compared to warfarin. Two studies, including the study of Sarrat et al. and the RENAL AF trial, were underpowered due to a smaller number of enrollees [23,30]. Two of the included studies collected data from National or Medicare databases; although they had a large study population they could not reliably comment on medication adherence and adequacy of treatment [18,28]. Finally, because of the complicated clinical picture, including high baseline bleeding and thrombosis risk, as well as the concomitant use of other medicines, including aspirin and antiplatelets in advanced CKD patients, any stroke, thrombosis, or bleeding event could not definitely be attributed to the type of therapy.

## Conclusions

Apixaban is a safe and reasonable alternative to warfarin in stages 4 and 5 of CKD and for patients on dialysis. In this population of patients, apixaban is as effective as warfarin in the prevention of stroke/systemic embolization and recurrent VTE in patients with non-valvular atrial fibrillation and arterial or venous thrombosis. Apixaban is also associated with reduced bleeding events than warfarin. As per the current evidence, apixaban's safety and efficacy profile are comparable and somewhat superior to warfarin in stages 4 and 5 CKD. However, more RCTs with higher powers and larger sample sizes are required to strengthen this evidence.
